# Strawberry *FaNAC2* Enhances Tolerance to Abiotic Stress by Regulating Proline Metabolism

**DOI:** 10.3390/plants9111417

**Published:** 2020-10-23

**Authors:** Jiahui Liang, Jing Zheng, Ze Wu, Hongqing Wang

**Affiliations:** 1 Department of Fruit Science, College of Horticulture, China Agricultural University, Beijing 100193, China; bs20173170791@cau.edu.cn (J.L.); sy20193172524@cau.edu.cn (J.Z.); 2 Key Laboratory of Landscaping Agriculture, Ministry of Agriculture and Rural Affairs, College of Horticulture, Nanjing Agricultural University, Nanjing 210095, China; wuze@njau.edu.cn

**Keywords:** strawberry, ATAF, *FaNAC2*, abiotic stress

## Abstract

The quality and yields of strawberry plants are seriously affected by abiotic stress every year. NAC (NAM, ATAF, CUC) transcription factors are plant-specific, having various functions in plant development and response to stress. In our study, *FaNAC2* from strawberry (*Fragaria × ananassa*, cultivar “Benihoppe”) was isolated and found to be a member of the ATAF sub-family, belonging to the NAC family of transcription factors. *FaNAC2* was strongly expressed in the shoot apical meristem and older leaves of strawberries, and was induced by cold, high salinity, and drought stress. To investigate how *FaNAC2* functions in plant responses to abiotic stress, transgenic *Nicotiana benthamiana* plants ectopically overexpressing *FaNAC2* were generated. The transgenic plants grew better under salt and cold stress, and, during simulated drought treatment, these transgenic lines not only grew better, but also showed higher seed germination rates than wild-type plants. Gene expression analysis revealed that key genes in proline biosynthesis pathways were up-regulated in *FaNAC2* overexpression lines, while its catabolic pathway genes were down-regulated and proline was accumulated more with the overexpression of *FaNAC2* after stress treatments. Furthermore, the gene expression of abscisic acid biosynthesis was also promoted. Our results demonstrate that *FaNAC2* plays an important positive role in response to different abiotic stresses and may be further utilized to improve the stress tolerance of strawberry plants.

## 1. Introduction

Agricultural crops grow in a constantly changing environment and are often subjected to abiotic stresses such as drought, heat, cold, and high salinity. These stresses are associated with increased accumulation of certain deleterious chemicals like reactive oxygen species (ROS), which affect the stability of cell membranes and the structure of proteins, finally leading to reduced crop yield and even death [1,2]. To adapt to environmental stress and complete their life cycle, plants have evolved a complex mechanism that tightly regulates gene expression through precise signaling. Until now, many stress-induced proteins including transcription factors (TFs), osmotic stress-adaptive proteins and key enzymes in abscisic acid (ABA) biosynthesis and signaling pathways have been reported [3,4,5].

The NAC [No apical meristem (NAM), Arabidopsis transcription activation factor (ATAF), and Cup-shaped cotyledon (CUC)] superfamily is one of the largest groups of plant-specific TFs, which not only play an important role in various stages of plant growth and development, but also participate in responses to biotic and abiotic stress [6,7,8,9,10]. Many NAC genes have been identified through their function in response to drought, cold, and high salinity stress [5]. Drought-induced genes *ANAC019*, *ANAC055*, and *RD26/ANAC072*, when overexpressed in Arabidopsis, improved drought tolerance [11,12]. Overexpression of *SlNAM1* from tomato could improve chilling tolerance of transgenic tobacco [13]. Besides, it has also been shown that *OsNAC6* (from rice), *SNAC2* (from rice), *TaNAC4* (from wheat), *TaNAC8* (from wheat), and *CarNAC1* (from chickpea), function as transcriptional activators in response to various abiotic stresses [9,14,15,16,17].

The ATAF TFs comprise a sub-family of NAC proteins. The first report of a stress-inducible ATAF-like gene was *StNAC* from potato [18]. Elicited *ATAF1* and *ATAF2* are considered to function as repressors of responsive genes under biotic and abiotic stress, because *ataf1* and *ataf2* mutants showed high stress resistance [19,20,21]. However, overexpression of *ATAF1* in Arabidopsis also exhibited enhanced plant tolerance to drought [22]. Subsequently, more ATAF family members from different species were explored in response to abiotic stress. *OsNAC52* from rice belongs to the ATAF sub-family, and functions as an important transcriptional activator in ABA-inducible gene expression [23]. *GmNAC2* plays a negative regulatory role in abiotic stress in *Glycine max*, and participates in the ROS signaling pathway by regulating the expression of ROS removal genes [24]. Overexpression of *SlNAC2* (from tomato) in Arabidopsis resulted in enhanced tolerance to salinity stress [25]. In addition, *SlNAC11* from tomato plays a stress-inducible TF role, depicting a positive response to abiotic stress tolerance [26]. *DgNAC1* from chrysanthemum worked as a positive regulator in responses to salt stress, as its overexpression in transgenic chrysanthemum showed lower levels of MDA (malondialdehyde) and reactive oxygen species (H_2_O_2_ and O^2−^), greater activities of SOD (superoxide dismutase), POD (peroxidase) and CAT (catalase), as well as more proline content than wild-type (WT) under salt stress [27]. In addition, it was reported that *CsATAF1* (from cucumber) was a crucial activator of the drought stress response via an ABA-dependent pathway, and inhibited ROS accumulation [28].

Strawberries are one of the most economically valuable crops in the world, which often suffers from water deficit, high salinity, flooding, and extreme temperature, leading to yield reduction [29,30,31]. Although many studies on responses of strawberry plants to abiotic stress have been reported, few data have showed NAC family members participating in abiotic stress resistance. Zhang et al. [31] showed that there are five *FvNAC* genes significantly contributing to various abiotic and biotic stress responses in woodland strawberry (*Fragaria vesca*), but their regulatory mechanisms are largely unknown.

In our study, *FaNAC2* was isolated from the cultivar strawberry (*Fragaria × ananassa*, cultivar “Benihoppe”); it was highly expressed in shoot apical meristem and old leaves of strawberry plants, and showed especially high expression in the guard cells of leaves. *FaNAC2* was induced under abiotic stress treatment, and many cis-acting elements that are responsive to abiotic stress were predicted in the *FaNAC2* promoter. We overexpressed *FaNAC2* in *Nicotiana benthamiana* (*N. benthamiana*), and found that the transgenic plants showed higher drought, cold and salt tolerance. These results indicated that *FaNAC2* might play a positive role in plant responses to abiotic stress.

## 2. Results

### 2.1. FaNAC2 Encodes an ATAF Protein that Belongs to the NAC Family

**Figure 1:**
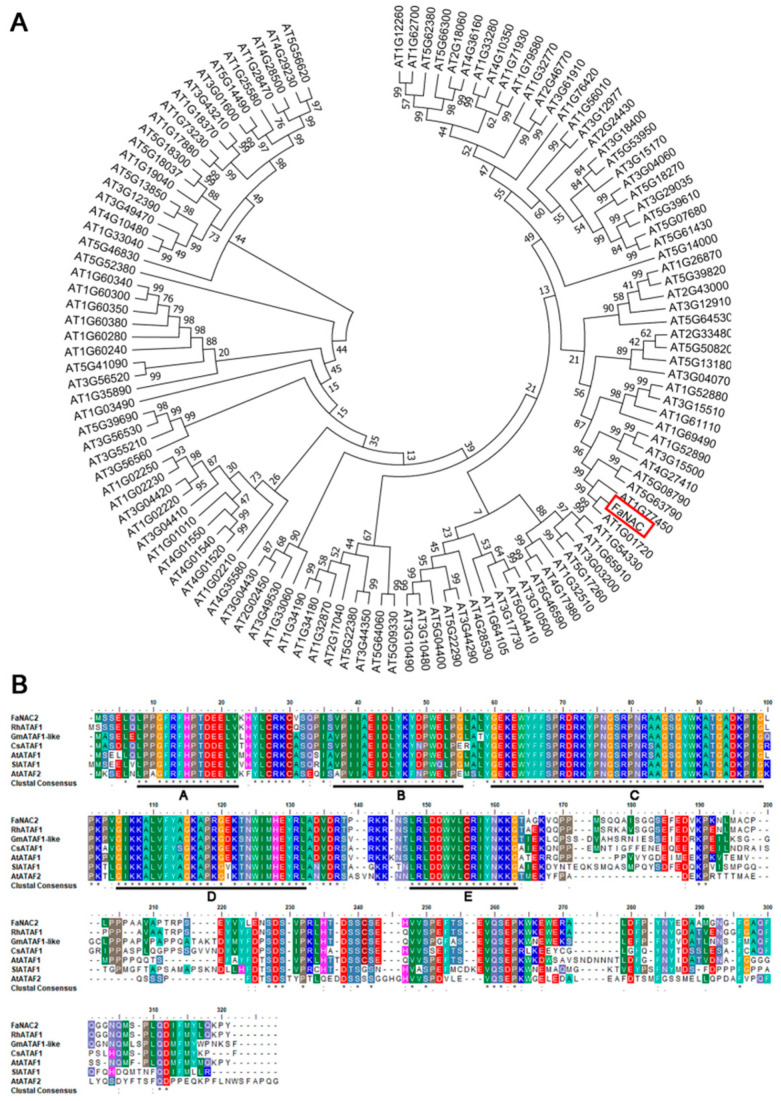
Phylogenetic analysis and amino acid sequence alignment of *FaNAC2*. (**A**) Phylogenetic relationship between *FaNAC2* (red boxed) from strawberry and NAC family members in Arabidopsis. MEGA 7.0 software was used to construct the Neighbor-Joining tree. The nearest *NAC* gene is *AT1G01720* (*AtATAF1* or *AtNAC2*). (**B**) Protein sequence alignment of ATAFs. The black underlines indicate the conserved N-terminal domain of NAC family. A–E represent five conserved sub-domains. Accession Numbers: Rosa hybrid cultivar, RhATAF1 (AXT99858.1); Arabidopsis thaliana, AtATAF1 (AT1G01720), AtATAF2 (AT5G08790); Suaeda liaotungensis K., SlNAC2 (JX860282.1); Glycine max, GmATAF1-like/GmNAC2 (AAX85979.1); Cucumis sativus L., CsATAF1 (Csa4M361820.1).

*FaNAC2* encodes a protein of 289 amino acids, and is a member of a plant-specific NAC family of transcription factors. We used the cDNA sequence of *FaNAC2* as a query to perform a BLAST search against 122 NAC family members of Arabidopsis, using MEGA 7.0 software. It was found that *FaNAC2* has the closest relationship with *AT1G01720*, which encodes an ATAF sub-group protein, and is also named *AtNAC2* (Figure 1A). A multiple sequence alignment of ATAF homologues from *Arabidopsis*, rose, *Suaeda liaotungensis*, soybean, cucumber and strawberry was performed. As shown in Figure 1B, high sequence similarities between *FaNAC2* and other plant ATAF proteins were found in the N-terminus, which contained several distinguishable conserved domains, and five sub-domains. These results indicated that *FaNAC2* encoded an ATAF1 protein and was a typical member of the NAC transcription factors.

### 2.2. Expression Pattern of FaNAC2

**Figure 2:**
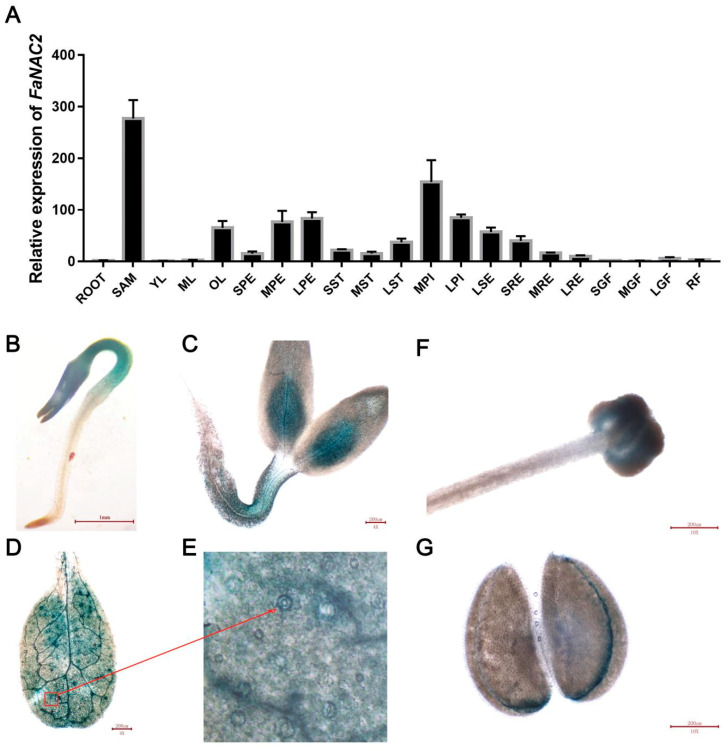
Expression pattern of *FaNAC2*. (**A**) qRT-PCR analysis of *FaNAC2* in different tissues of strawberry. ROOT: roots; SAM: shoot apical meristem; YL: young leaves (the second folded leaf); ML: mature leaves (the third or fourth fully expanded leaf); OL: old leaves (below the seventh leaf); SPE: petals from small flower buds (length < 0.5 cm); MPE: petals from middle flower buds (length 0.5–0.8 cm); LPE: petals from large opened flower; SST: stamen from small flower buds; MST: stamen from middle flower buds; LST: stamen from large opened flower; MPI: pistils from middle flower buds; LPI: pistils from large flower buds; LSE: sepals from large opened flower; SRE: receptacles from small flower buds; MRE: receptacles from middle flower buds; LRE: receptacles from large opened flower; SGF: small green fruits; MGF: middle green fruits; LGF: large green fruits; RF: red fruits. The *FaACTIN* gene was used as an internal reference to normalize the expression data. Data are presented as averages of three biological repeats. Bars are means (±S.D.) of three independent experiments. (**B**,**C**) β-glucuronidase (GUS) analysis of 5 DAG (days after germination) Pro*FaNAC2*–GUS *N. benthamiana (Nicotiana benthamiana)* seedlings. (**D**,**E**) GUS analysis of true leaf of 13 DAG seedling and its enlarged view. The red arrow points to guard cells in (E). (**F**,**G**) GUS analysis of stigma and anther of 35 DAG Pro*FaNAC2*–GUS lines after planting on soil.

To explore the function of *FaNAC2*, we first analyzed its spatial and temporal expression patterns in strawberry plants. The qRT-PCR (quantitative RT-PCR) results showed that *FaNAC2* was expressed at higher levels in shoot apical meristem, old leaves and flowers, compared to roots and fruits (Figure 2A). For different stages of leaves, the expression of *FaNAC2* in older leaves was more than that in younger leaves and mature leaves, indicating that the accumulation of *FaNAC2* might increase with the senescence of leaves. Amongst the different sizes of flower buds, *FaNAC2* had a high level of expression in the late petals and early stages of pistils (Figure 2A).

To better understand the expression pattern of *FaNAC2*, the transgenic *N. benthamiana* contained a *β-glucuronidase* (*GUS*) reporter gene which, under the control of *FaNAC2* promoter, was produced and detected. The results revealed that the GUS signals were detected in cotyledons and true leaves of young plants (Figure 2B,C), and it showed strong expression in the guard cells of true leaves (Figure 2D,E), suggesting that *FaNAC2* might have a function in controlling stoma. In addition, *GUS* expression was also be seen in the stigmas and anthers of the flowers (Figure 2F,G).

### 2.3. FaNAC2 Is Induced by Cold, Salinity, and Drought Treatment

**Figure 3:**
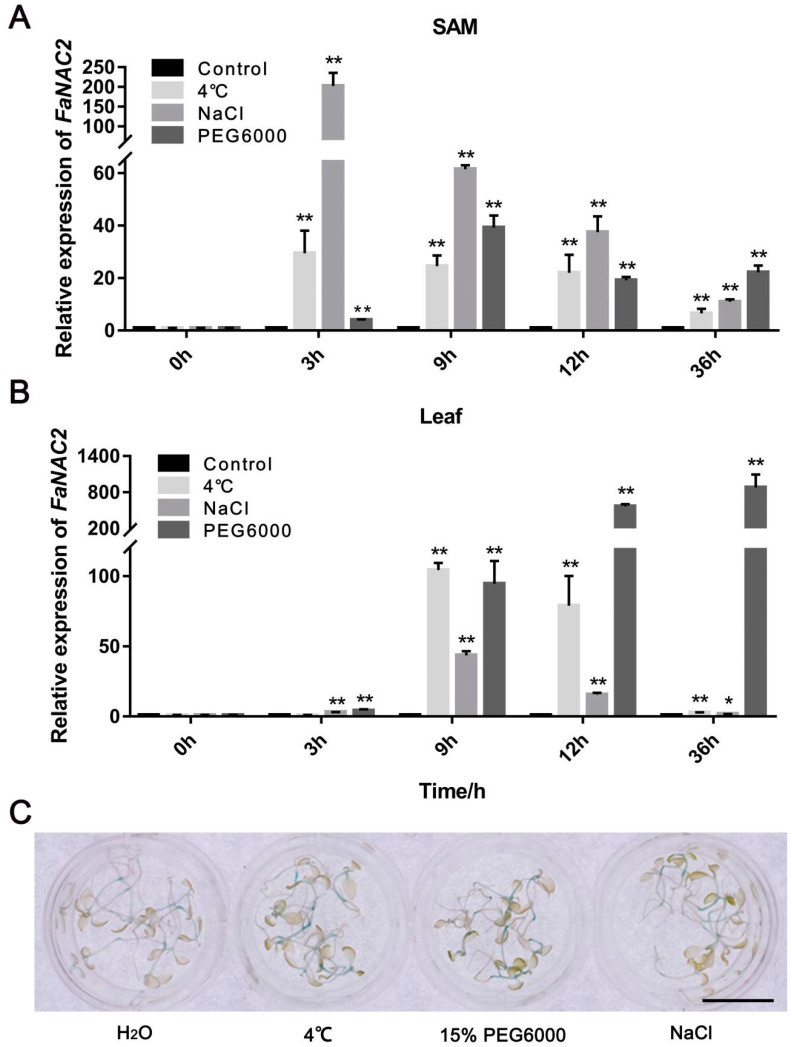
*FaNAC2* is induced by cold, drought, and salinity stress. (**A**) The expression level of *FaNAC2* in the SAM (shoot apical meristem) of strawberry under different abiotic stresses. (**B**) The expression level of *FaNAC2* in the leaves of strawberry under different abiotic stresses. (**C**) Different abiotic stress treatments to 7 DAG (days after germination) Pro*FaNAC2*–GUS seedlings. Salinity treatment used 200 mM NaCl. Three independent experiments were performed and error bars indicate standard deviation (Student’s *t*–test; * *p* < 0.05; ** *p* < 0.01). The scale bar represents 1 cm.

Since many *cis*-elements related to abiotic stress, such as ABRE (response to abscisic acid), LTR (response to cold) and MBS (response to drought), were found on the promoter sequence of *FaNAC2* (Table 1), we hypothesized that *FaNAC2* would also respond to abiotic stress. Thus, we performed stress treatments, including 200 mM NaCl, 20% polyethylene glycol (PEG) 6000, and 4 °C, to detect the expression changes of *FaNAC2* using 3 MAC (months after cutting node bud from runners) tissue cultured seedlings of strawberry for qRT-PCR assay. The results revealed that its expression was strongly up-regulated in both SAM (shoot apical meristem) and leaves in response to abiotic stress (Figure 3A,B). During cold and salt treatment, the expression of *FaNAC2* in the SAM was induced at 3 h and then gradually decreased compared to the initial expression levels, while the expression of *FaNAC2* from the leaves was a little slower, being induced only at 6 h following stress. Under the condition of simulated drought treatment, *FaNAC2* showed a great difference in expression pattern in different tissues; although *FaNAC2* was induced at 9 h, the expression of *FaNAC2* in the SAM was gradually decreased from 12 h after induction, while *FaNAC2* expression in the leaves increased at all the times tested, which indicated that *FaNAC2* might participate in drought stress response, mainly in the leaves.

To confirm the expression pattern, different stress treatments were performed at 7 DAG (days after germination) of Pro*FaNAC2*–GUS *N. benthamiana*, and the results showed that the *GUS* expression became stronger under cold, simulated drought and salt stress, indicating that *FaNAC2* could be induced by different abiotic stresses (Figure 3C). Taking all these results together, it could be inferred that *FaNAC2* might function in different tissues to cope with different stress conditions.

### 2.4. Overexpression of FaNAC2 Improves Stress Tolerance in Transgenic N. benthamiana

**Figure 4:**
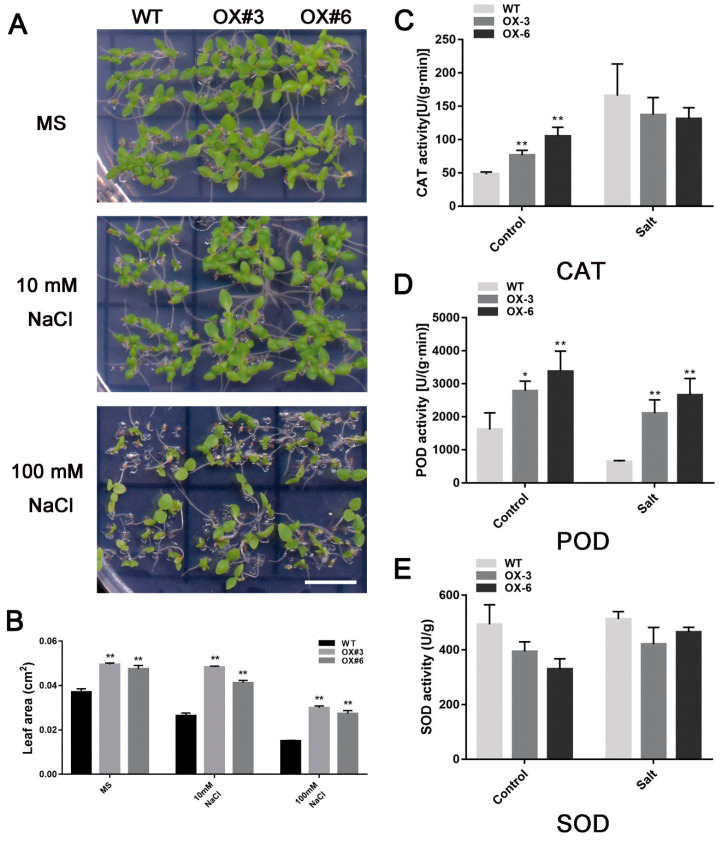
Comparison of resistance to salinity of *35S::FaNAC2* and wild-type (WT) lines. (**A**) Analysis of 13 DAG seedlings of *35S::FaNAC2* and WT seeds under salt and control treatments. OX: overexpression. (**B**) Leaf area of *35S::FaNAC2* and WT 13 DAG seedlings under salt and control treatments. (**C**–**E**) Enzyme activity of several enzymes related to plant resistance to salt. CAT: Catalase; POD: Peroxidase; SOD: Superoxide dismutase. The scale bar represents 1 cm. Bars are means (±S.D.) of three independent experiments (Student’s *t*–test; * *p* < 0.05; ** *p* < 0.01).

To further investigate how *FaNAC2* plays roles in abiotic stress resistance, *FaNAC2* was ectopically transformed into *N. benthamiana* under the control of a CaMV (Cauliflower Mosaic Virus)–35S promoter, and ten positive lines were obtained through screening using kanamycin and RT-PCR analysis. By observing the growth potential of germination of 13 DAG *35S::FaNAC2* and wild-type lines under salt treatments, we found that *35S::FaNAC2* lines exhibited better growth under salt stress; for example, the leaf areas of transgenic plants were larger than WT following salt treatments (Figure 4A,B), indicating that overexpression of *FaNAC2* promoted the salt tolerance of plants.

As the plants get stronger, the 40 DAG *35S::FaNAC2* lines and WT plants were irrigated with 300 mM NaCl for one week, with normal watering as a control. By comparing the enzyme activity of antioxidant enzymes including catalase (CAT), peroxidase (POD) and superoxide dismutase (SOD) from two lines under different conditions, it was found that CAT enzyme activity in *35S::FaNAC2* lines was significantly higher than that in WT lines under control conditions, but there was no significant difference after salt treatment (Figure 4C). POD activity of *35S::FaNAC2* plants was higher than that of WT lines under both control and salt treatment, while there was no significant change in SOD enzyme activity (Figure 4D,E). These results suggested that *FaNAC2* might promote plant salt tolerance by partially affecting the activity of some antioxidant enzymes.

**Figure 5:**
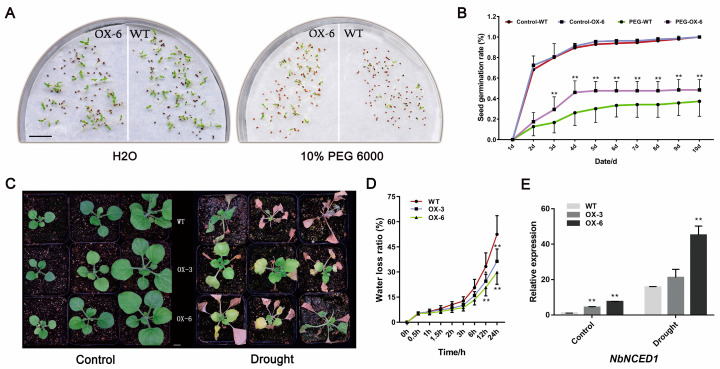
Comparison the rehydration rate of *35S::FaNAC2* and WT lines under drought stress. (**A**,**B**) Germination rates of *35S::FaNAC2* and WT seeds under water and simulated drought conditions. (**C**) Phenotypes of 40 DAG *35S::FaNAC2* and WT lines under control and rehydration conditions. (**D**) Water loss rate (WLR) of leaves from *35S::FaNAC2* and WT lines. (**E**) The qRT-PCR analysis of *NbNCED1* expression, related to ABA biosynthesis pathways. The scale bar represents 1 cm. Bars are means (± S.D.) of three independent experiments (Student’s *t*-test; * *p* < 0.05; ** *p* < 0.01).

Through different treatments for seed germination, we found that there was no difference in seed germination rates between *35S::FaNAC2* and WT lines under normal conditions. However, the seeds of *35S::FaNAC2* lines had a 10% higher germination rates than that of WT plants under the treatment of simulated drought (10% PEG 6000; Figure 5A,B). Subsequently, we conducted drought treatment on 40 DAG *35S::FaNAC2* and WT plants; *35S::FaNAC2* plants had a higher recovery rate after rehydration (Figure 5C). Comparing the WLR (water loss rate) from leaves of the two lines, it was found that the dryness of *35S::FaNAC2* leaves was lower, suggesting that they retained leaf water more easily (Figure 5D). Besides, a key gene for ABA biosynthesis, *NbNCED1* (*9-cis-epoxycarotenoid dioxygenase 1*), was also detected in both the normal and drought treatment conditions, and *NbNCED1* expression in *35S::FaNAC2* lines was significantly higher than that of WT (Figure 5E). Taking all these results together, our data suggested that *FaNAC2* might exist as a positive regulator of drought stress tolerance.

**Figure 6:**
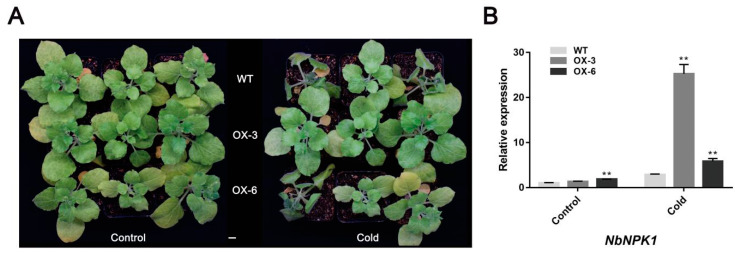
Comparison of *35S::FaNAC2* and WT plants under cold stress. (**A**) Phenotypes of *35S::FaNAC2* lines and WT under control and cold conditions. (**B**) Expression of *NbNPK1* related to plant resistance to cold pathways in *35S::FaNAC2* lines and WT under cold treatment. The scale bar represents 1 cm. Bars are means (±S.D.) of three independent experiments (Student’s *t*-test; ** *p* < 0.01).

In order to understand the function of *FaNAC2* in cold stress, *35S::FaNAC2* lines and WT plants were subjected to cold treatment, which was performed as follows: 4 °C for 2 h, 0 °C for 1 h, −5 °C for 1 h and 4 °C for 1 h. The results showed that *35S::FaNAC2* lines were more cold-resistant than WT plants, showing less damage (Figure 6A). Meanwhile, a key gene, *NbNPK1* (*Nicotiana protein kinase 1*), which was involved in cold resistance signal transmission in plants, was found to show increased expression in *35S::FaNAC2* lines compared to WT plants under control treatment, while it was significantly higher than that in WT plants under cold treatment (Figure 6B). All of these results demonstrated that *FaNAC2* might play a positive role in response to abiotic stress.

### 2.5. FaNAC2 Promotes Plant Abiotic Stress Tolerance via Regulating Proline Metabolism

**Figure 7:**
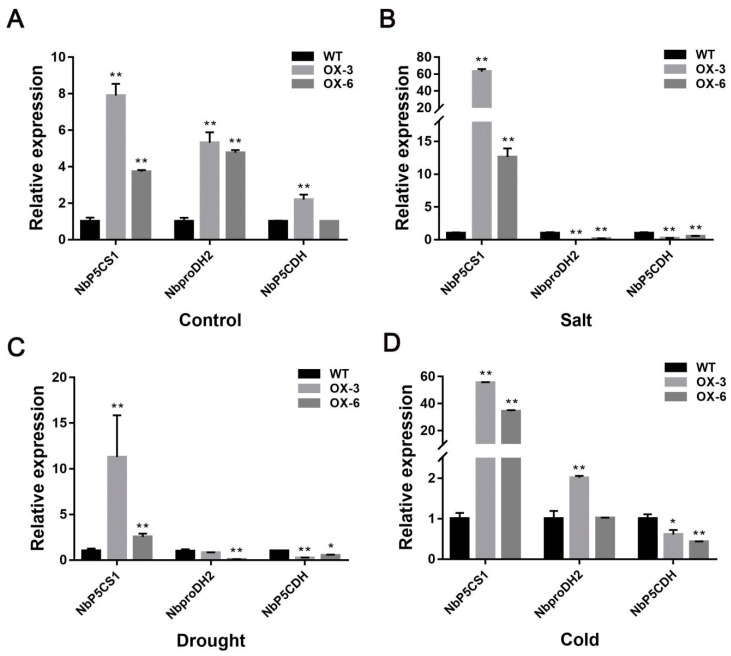
The qRT-PCR analysis of several genes related to proline matabolism in *35S::FaNAC2* lines and WT under different abiotic stress treatments. (**A**) Expression of genes in *35S::FaNAC2* lines and WT under control conditions. (**B**) Expression of genes in *35S::FaNAC2* lines and WT under salt stress. (**C**) Expression of genes in *35S::FaNAC2* lines and WT under drought stress. (**D**) Expression of genes in *35S::FaNAC2* lines and WT under cold stress. Bars are means (±S.D.) of three biological replicantes experiments (Student’s *t*-test; * *p* < 0.05; ** *p* < 0.01).

To further investigate how *FaNAC2* regulates plant stress tolerance, we detected the expression changes of key genes in proline biosynthesis, *NbP5CS1* (*Pyrroline-5 carboxylate synthetase 1*), and catabolism, *NbP5CDH* (*P5C dehydrogenase*) and *NbproDH2* (*Proline dehydrogenase 2*) from *35S::FaNAC2* and WT plants (Figure 7). Compared with WT, *NbP5CS1* expression from *35S::FaNAC2* lines was increased either in control or salt stress conditions (Figure 7A,B). *NbproDH2* expression was also higher in transgenic lines than WT in control conditions, while its expression significantly decreased after salt treatment. The expression of another key gene in the proline catabolism pathway, *NbP5CDH*, in *35S::FaNAC2* lines was also down-regulated after salt treatment (Figure 7B).

Following drought treatment, the expression of *NbP5CS1* in *35S::FaNAC2* was significantly higher than that of WT, while the expression of *NbP5CDH* and *NbproDH2* was decreased compared with that of WT (Figure 7C). 

Similar to the two previous stress treatments, further analysis revealed that the expression of *NbP5CS1* was up-regulated in *35S::FaNAC2* lines under both control and cold treatment. The *NbproDH2* expression of *35S::FaNAC2* lines was more than five times as high as that in WT plants under the control treatment, whereas it was twice as high as that in WT plants under the cold stress, suggesting that *NbproDH2* of *35S::FaNAC2* was decreased during the cold resistance compared to control condition. Meanwhile, *NbP5CDH* expression was relatively high in the control treatment, but lower in the WT plants after cold treatment (Figure 7D).

**Figure 8:**
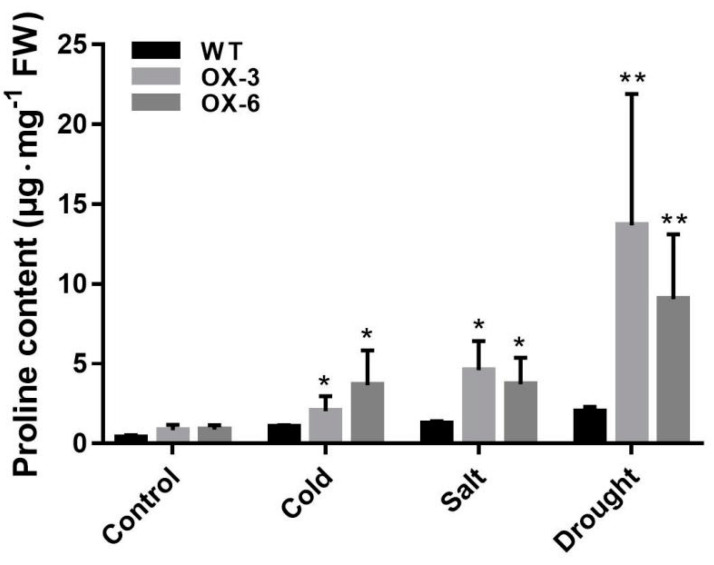
The proline content in *35S::FaNAC2* lines and WT leaves under different abiotic stress treatments. The fifth leaf was collected for proline content determination. Bars are means (±S.D.) of three independent experiments (Student’s *t*-test; * *p* < 0.05; ** *p* < 0.01).

In addition, proline levels were detected under control and abiotic stress. It was found that proline content in *35S::FaNAC2* lines was slightly higher than WT plants in the control environment. With different abiotic stress treatments on the plants, proline content in *35S::FaNAC2* strains was significantly higher than WT strains (Figure 8). Due to both the proline biosynthesis gene and the proline catabolism gene being up-regulated under normal conditions, it was speculated that *FaNAC2* might promote proline metabolism to sustain proline hemostasis for normal growth of transgenic plants, but *FaNAC2* could improve proline levels, in response to different abiotic stresses, for increased tolerance.

Taking all of these results into account, we concluded that, in general, *FaNAC2* might be involved in plant abiotic stress tolerance by regulating proline accumulation and catabolism.

## 3. Discussion

### 3.1. The Expression Pattern of FaNAC2

Many NAC family members have been reported to be involved in plant growth and development processes, such as SAM (shoot apical meristem) establishment, lateral root development, leaf senescence and cell wall formation [32,33,34,35]. Thus, we speculated that *FaNAC2* may also be involved in many plant developmental processes. Our data show that *FaNAC2* might be involved in leaf senescence, as it expressed highly in old leaves (Figure 2A). These results are similar to the function of other members of the NAC family. Overexpression of *OsNAC2* has been shown to promote leaf senescence via ABA biosynthesis [36], while ABA biosynthesis was also activated in the *35S::FaNAC2 N. benthamiana* lines as our data shown (Figure 5), thus, whether *FaNAC2* promotes leaf senescence via ABA synthesis needs further investigation.

GUS analysis showed high levels of *FaNAC2* promoter activity in guard cells of transgenic *N. benthamiana* leaves (Figure 2D,E). Considering these results, it is possible that there is a function for *FaNAC2* in plant development. Stomata can affect transpiration and photosynthesis by regulating their closure with the changing environment, via sensing ABA signals under adverse conditions [37,38,39,40]. Thus, *FaNAC2* may regulate transpiration and leaf water retention by adjusting guard cells. Besides, although the theory that root-sourced ABA can act as a signal to regulate stomatal aperture gained widespread acceptance, ABA biosynthetic mutants showed that stomatal aperture is predominantly regulated by leaf-sourced ABA [41,42,43,44]. In our results (Figure 5E), *FaNAC2* could promote the key gene in ABA synthesis pathways in the leaf, thus we speculated that *FaNAC2* may regulate stomatal closure by participating in ABA biosynthesis in leaves, further improving the drought resistance of plants.

### 3.2. FaNAC2 Functions as a Positive Regulator in Response to Abiotic Stress

Abiotic stress is an important factor that threatens the yield and quality of strawberry. To alleviate the damage of abiotic stress, plants usually initiate complex adaptation via genetic mechanisms including regulation of gene expression and increased concentration of osmolytes [5,27]. There are several reports revealing that the ATAF sub-group of TFs belonging to the NAC family play important roles in response to abiotic stress; however, their function is still under debate. In Arabidopsis, ATAF1 was reported to negatively regulate stress-responsive gene expression during drought stress, because *ataf1* mutants displayed higher recovery rates than WT under drought [20]. Overexpression of *GmNAC2* reduces abiotic stress tolerance in *Glycine max*, which also functions as a negative regulator by participating in ROS signaling pathways [24]. It has been reported that *AtATAF1* overexpression in transgenic lines enhanced drought tolerance [22]. Subsequently, *OsNAC52* from rice, *SlNAC2* from tomato, *DgNAC1* from chrysanthemum and *CsATAF1* from cucumber were reported to function as positive regulators in response to abiotic stress [23,25,27,28].

In our study, GUS analysis showed that the promoter activity of strawberry *FaNAC2* was induced by drought, salt and cold stress (Figure 3C), and *FaNAC2* expression levels exhibited the same trend that was verified by qRT-PCR (Figure 3A,B) in strawberry. Besides, ectopic overexpression of *FaNAC2* in *N. benthamiana* plants showed higher tolerance to salinity, drought and cold stress (Figure 4, Figure 5 and Figure 6). Members of the ATAF sub-family in dicotyledons have conserved domains and can be identified by some conserved regions that respond positively to stress. Overexpressed transgenic lines of *ANAC019*, *ANAC055* and *ANAC072* improved the drought resistance of plants, and the conserved *cis*-elements CATGT and CACG, for their binding, were identified [12]. However, the central function of *FaNAC2* in response to stress in strawberry is still unknown, and further studies on the abiotic stress pathway involving *FaNAC2* are needed.

Proline accumulation has been reported to occur after biotic and abiotic stress [45,46,47]. It varies across different species under stress and can be more than 100 times higher than that under control conditions [48]. In our study, both the synthesis and catabolism pathways of proline were induced in *35S::FaNAC2* lines (Figure 7A), suggesting that the overexpression of *FaNAC2* can promote the metabolism of proline. However, in a stress environment, the expression levels of *NbP5CS1* in *35S::FaNAC2* were still higher than that in WT, and both *NbproDH2* and *NbP5CDH* were decreased compared to control condition (Figure 7B–D). Further, the proline content of 35S::*FaNAC2* was higher than WT under the abiotic stress condition (Figure 8). We speculate that *FaNAC2* might promote the accumulation of proline under adverse conditions by activating proline synthesis and inhibiting proline degradation, so as to promote the stress tolerance of plants.

Taken together, *FaNAC2* from strawberry might play a positive role in response to abiotic stress by regulating proline metabolism. Although the role of *FaNAC2* in stress tolerance needs to be further validated in strawberries, we demonstrate that it can serve as a candidate gene to enhance stress tolerance, as long as the spatial–temporal expression is controlled.

## 4. Materials and Methods

### 4.1. Plant Materials, Growth Conditions

The strawberry cultivar “Benihoppe” (*Fragaria × ananassa* Duch.) was used in this study and maintained in a plant culture room (23 ± 1 °C, relative humidity of 40%, 16 h/8 h light/dark cycles). The tissue cultured seedlings were initiated from node bud of runners, which were collected from actively growing plants and disinfected with 70% ethanol (30 s) and 1% NaClO (10 min).

Most of *N. benthamiana* seeds were germinated on MS solid medium with 20% sucrose and grown for 13 days. Then, the seedlings were transplanted to the soil and grown in the culture room. For *N. benthamiana* seeds treated by simulate drought, the seeds were simply spread flat on a filter paper soaked in water or 10% PEG 6000.

### 4.2. Gene Isolation and Sequence Alignment

Total RNA samples were extracted from collected shoot apicals and leaves using an E.Z.N.A Total RNA Kit (Omega., Norcross, Georgia, USA). HIScript II Reverse Transcriptase (Vazyme, Nanjing, China) was used for cDNA synthesis. The primers of *FaNAC2* were designed according to the GDR Database (Genome Database for Rosaceae) [49]. The CDS (coding sequence) of *FaNAC2* was obtained from the cDNA of “Benihoppe”. Phylogenetic analysis was performed using MEGA version 7 (http://www.megasoftware.net/) [50]. Alignments of the *FaNAC2* full-length amino acid sequence with ATAF homologues from other species were performed using BioEdit software (http://www.mbio.ncsu.edu/BioEdit/bioedit.html) and ClustalW for multiple sequence alignments (http://www.ch.embnet.org/software/ClustalW.html).

### 4.3. Promoter Isolation, Prediction of Cis-Elements and GUS Activity Assay

Genomic DNA was extracted from strawberry “Benihoppe” using a TIANquick Midi Purification Kit (TianGen., Beijing, China). The primers for the promoter of *FaNAC2* were designed using the sequence from the GDR database and the sequence of the promoter was obtained using the DNA of “Benihoppe” strawberry as template, then the fragment was proofread and sequenced. *FaNAC2* promoter was cloned into the pCAMBIA1391 vector using the TrelisfTM SoSoo cloning Kit (TsingKe, Beijing, China) to generate the reporter construct pCAMBIA1391–Pro*FaNAC2*–GUS. The primers used are listed in Table S1. The construct was stably transformed into *N. benthamiana* by *Agrobacterium*-mediated transformation, as described below. Prediction of *cis*-elements was performed using the Plantcare online tool (http://bioinformatics.psb.ugent.be/webtools/plantcare/html/).

For GUS analysis, samples were incubated with GUS staining buffer (including 2 M ferri/ferrocyanide, 0.1% Triton X-100, 0.1 M sodium phosphate buffer, 0.5 mg·mL^−1^ X-gluc, pH 7) at 37 °C for 9 h, then stained samples were decolorized using 75% ethanol.

### 4.4. Gene Expression Analysis

qRT-PCR was employed to detect the expression of different genes. The qRT-PCR reactions (20 µL volume containing 500 ng cDNA as template) were run using SYBR Premix ExTaq (TAKARA., Beijing, China) as enzyme and ABI QuantStudio™ 6 Flex PCR System (ABI., New York, NY, USA). The 2^−ΔΔCT^ method was used for qRT-PCR analysis. *FaACTIN* and *NbACTIN* were used as internal controls for gene expression. The qRT-PCR primers of *NbNCED1*/*NbNPK1*/*Nb5CS1*/*NbproDH2*/*NbP5CDH* for qRT-PCR were designed according to the *N. benthamiana* database [51]. The template for analyzing the expression of these genes was cDNA of the fifth tobacco leaves from different treatments. PCR was performed in triplicate using RNA samples extracted from three independent plants. Each reaction was performed using three biological replicates and verified by melting curve analysis. The primers are listed in Table S1.

### 4.5. Stable Transformation of N. benthamiana

For overexpression of *FaNAC2* in *N. benthamiana*, the vector pCAMBIA2300 with kanamycin resistance was used for stable transformation. The *FaNAC2* ORF was inserted into pCAMBIA2300 using the TrelisfTM SoSoo cloning Kit (TsingKe) and driven by a CaMV 35S promoter. The *35S::FaHAN* was introduced into *Agrobacterium tumefaciens* strain GV3101 and then transformed into *N. benthamiana* leaf dishes by the method of *Agrobacterium*-mediated transformation [52]. The obtained kanamycin-resistant plants were screened again by RT-PCR. The PCR mix was from TsingKe and the PCR primers used for vector construction are listed in Table S1.

### 4.6. Abiotic Stress Treatment

The 3-months-old “Benihoppe” strawberry tissue cultured seedlings, which were obtained from the node bud of runners, were used for stress treatment. Three simulated stress conditions of 200 mM NaCl, 20% PEG 6000 and 4 °C were set, and MS liquid medium was used as a control to treat for 0 h, 3 h, 6 h, 12 h and 36 h. The qRT-PCR was performed in biological triplicate using RNA samples extracted from three independent plants. For GUS staining, the abiotic stress treatment for 7 DAG (days after germination) *N. benthamiana* seedlings was 200 mM NaCl, 15% PEG 6000 and 4 °C; the treatment lasted 12 h.

Regarding abiotic stress treatment of *35S::FaNAC2* transgenic *N. benthamiana* lines and WT plants, simulated drought treatment for seeds was used with 10% PEG 6000; drought treatment of 40 DAG seedlings was conducted by stopping the watering for 20 d, until the leaves were all wilted, then re-watering for 5 d to observe the recovery of plants. Cold treatment was performed to put the plants under normal indoor conditions after 4 °C for 2 h, 0 °C for 1 h, −5 °C for 1 h and 4 °C for 1 h, and the status of the plants was observed. Salt treatment for seedings of *35S::FaNAC2* and wild-type lines were MS solid medium with 10 mM NaCl and 100 mM NaCl, respectively. Considering that 40 DAG seedlings were stronger, salt treatment was enhanced to 300 mM NaCl. These experiments were repeated three times and each line had three biological replicates.

### 4.7. Determination of SOD, POD and CAT Enzyme Activities

Approximately 0.15 g of the fifth expanded leaf from each line (including *35S::FaNAC2* and WT lines) was homogenized in 5 mL pre-cooled Phosphate buffered saline, at 4000 rpm for 10 min at 4 °C, then 2 mL of supernatant was drained and placed on ice for determination of different enzyme activities, according to measurements as follows.

CAT (Catalase) activity was measured spectrophotometrically at 240 nm [53]. The reaction mixture contained 100 mM sodium phosphate buffer (pH 7.0), 30 mM H_2_O_2_ and 100 μL of crude extract in a total volume of 3 mL. The absorbance was read quickly every 1 min, for a total of 4 min.

The activity of POD (peroxidase) was determined at 420 nm using a spectrophotometer, with callus lignin as substrate. The reaction mixture contained 100 mM sodium phosphate buffer (pH 6.0), 5 mM hydrogen peroxide, 5 mM guaiacol and 100 μL crude extract, with a total volume of 3 mL, at room temperature (±25 °C) [54].

The activity of SOD (superoxide dismutase) was determined by inhibiting the photoreduction of SOD to NBT (nitro-blue tetrazolium) [55]. The reaction mixture contained a final volume of 3 mL of 50 mM sodium phosphate buffer (pH 7.6), 0.1 mM Ethylene Diamine Tetraacetic Acid (EDTA)-Na_2_, 50 mM sodium carbonate, 12 mM L-methionine, 50 mM NBT, 10 µM riboflavin and 100 µL of crude extract. Then, the reaction mixture was exposed to white light for 30 min for the SOD reaction. After incubation, the absorbance was recorded at 560 nm with a spectrophotometer.

### 4.8. Determination of WLR (Water Loss Rate) in N. benthamiana Leaves

The fifth leaf was collected from half-month-old tobacco plants which were grown in the soil and dried naturally on filter paper. The temperature was 25 °C, the relative humidity was 38%, and weight measurements were taken at 0 h, 0.5 h, 1 h, 1.5 h, 2 h, 3 h, and 6 h. This experiment was repeated three times to calculate the weight and rate of water loss in each period.

### 4.9. Proline Measurement

To determine free proline level, 0.1 g of fourth expanded leaf samples from each line (including *35S::FaNAC2* and WT lines) was homogenized in 3% (*w*/*v*) sulphosalycylic acid and then homogenate filtered through filter paper [56]. The mixture was heated at 100 °C for 30 min in a water bath after the addition of acid ninhydrin and glacial acetic acid. The reaction was then stopped by ice bath. The mixture was extracted with toluene and the absorbance of the fraction with toluene aspired from the liquid phase was read at 520 nm. Proline concentration was determined using a calibration curve.

### 4.10. Statistical Analysis

Microsoft Excel 2019 (Microsoft Corp., Redmond, Washington, USA) and GraphPad (GraphPad Software Inc., San Diego, California, USA) were used for analyzing the experimental data. Data for *p*-values were analyzed by Student’s *t* test at a significance level of 0.05 or 0.01. Comparisons between multiple samples were determined using Tukey’s multiple comparisons test.

## Figures and Tables

**Table 1:** Predicted *cis*-elements in the promoter of *FaNAC2*.

*Cis*-elements	Sequence	Number	Character
ABRE	ACGTG	4 (+)	Response to abscisic acid
ARE	AAACCA	1 (+)	Response to anaerobic process
Box-4	ATTAAT	1 (+)	Response to light
CGTCA-motif	CGTCA	3 (+)	Response to Jasmonic Acid
G-box	CACGTG	7 (+)	Response to light reaction
GCN4-motif	TGAGTCA	1 (+)	Involved in endosperm expression
LTR	CCGAAA	1 (+)	Response to cold
MBS	CAACTG	1 (+)	Response to drought
RY-element	CATGCATG	1 (+)	Specific seeds’ regulation
TCA-element	TCAGAAGAGG	2 (+)	Response to salicylic acid
TGA-element	AACGAC	2 (+)	Response to auxin

## Supplementary Materials

The following are available online at https://www.mdpi.com/2223-7747/9/11/1417/s1, Table S1: Primers used in this study.
