# Supplementary material for: Strawberry FaNAC2 Enhances Tolerance to Abiotic Stress by Regulating Proline Metabolism
Source: Plants (Basel). 2020 Oct 23;9(11):1417. doi: 10.3390/plants9111417 (PMC7690739; doi:10.3390/plants9111417)
Supplement: Supplementary file 1 [file plants-09-01417-s001.pdf]

Table S1. Primers used in this study.

**Primer Names**	**Primer Sequences (5′-3′)**
**Primers used for isolating genes**	
FaNAC2-F	ATGTCGTCGGAGTTACAGTTAC
FaNAC2-R	TCAATACGGCTTCTGCAGGTAC
**Primers used for cloning gene promoter and its analysis**	
ProFaNAC2-F	GAGCGCATGTTTATGTGGCTAG
ProFaNAC2-R	CACCGGCGGTAATCTTGTTTTTAC
pCAMBIA1391-ProFaHAN-F	CGACGGATCCCCGGGAATTCCAGCTCATTAGGGCTAACG
pCAMBIA1391-ProFaHAN-R	GGACTCCTCTTAGAATTCAGGAAAGGCGTGGGTG
**Primers used for construction of vectors for gene overexpression**	
pCAMBIA2300-FaNAC2-F	GAGGACAGGGTACCCGGGATGTCGTCGGAGTTACAGTTAC
pCAMBIA2300-FaNAC2-R	CTCTAGAGGATCCCCGGGTCAATACGGCTTCTGCAGG
**Primers used in qRT-PCR**	
QRT-FaNAC2-F	CAGACTCGAGCTGCTCGGAG
QRT-FaNAC2-R	GCAGCGGCGACATCTGATTC
QRT-FaACTIN-F	TGGGTTTGCTGGAGATGAT
QRT-FaACTIN-R	CAGTTAGGAGAACTGGGTGC
QRT-NbACTIN-F	TGGCTCTTGACTACGAGCAGGAGCTT
QRT-NbACTIN-R	ACCACTGAGCACAATGTTACCGTAGAGGT
NbP5CS1-qRT-F	CCAGTGGCTTCGCTGTCGAT
NbP5CS1-qRT-R	CCTGAAGCCGCCTGGAACAT
NbproDH2-qRT-F	CGCTGACGCTACAGATGGCA
NbproDH2-qRT-R	CCGCCGTCCGATAGTTTGCT
NbP5CDH-qRT-F	ACCGCGCATGACTCTCTTCA
NbP5CDH-qRT-R	TCGGGCCCAAGGATCTTCCA
NbNCED1-qRT-F	TAACGGCGCTAACCCGCTTT
NbNCED1-qRT-R	GAACCATGCCGTCACCGTCA
NbNPK1-qRT-F	CTCCGTTCGCCGATCTCTGG
NbNPK1-qRT-R	GCGGGCAGCTCAGCTTTAGA
